# The indirect NMDAR inhibitor flupirtine induces sustained post-ischemic recovery, neuroprotection and angioneurogenesis

**DOI:** 10.18632/oncotarget.4226

**Published:** 2015-05-22

**Authors:** Hanna M. Jaeger, Jens R. Pehlke, Britta Kaltwasser, Ertugrul Kilic, Mathias Bähr, Dirk M. Hermann, Thorsten R. Doeppner

**Affiliations:** ^1^ University of Duisburg-Essen Medical School, Department of Neurology, Essen, Germany; ^2^ LWL-Klinik Muenster, Department of Addiction Disorders, Muenster, Germany; ^3^ Istanbul Medipol University, Regenerative and Restorative Medical Research Center, Istanbul, Turkey; ^4^ University of Goettingen Medical School, Department of Neurology, Goettingen, Germany

**Keywords:** pathology, excitotoxicity, focal cerebral ischemia, neuroprotection, N-methyl-D-aspartate-receptors (NMDAR)

## Abstract

N-methyl-D-aspartate receptor (NMDAR) activation induces excitotoxicity, contributing to post-stroke brain injury. Hitherto, NMDAR deactivation failed in clinical trials due to insufficient pre-clinical study designs and drug toxicity. Flupirtine is an indirect NMDAR antagonist being used as analgesic in patients. Taking into account its tolerability profile, we evaluated effects of flupirtine on post-stroke tissue survival, neurological recovery and brain remodeling. Mice were exposed to stroke and intraperitoneally treated with saline (control) or flupirtine at various doses (1-10 mg/kg) and time-points (0-12 hours). Tissue survival and cell signaling were studied on day 2, whereas neurological recovery and tissue remodeling were analyzed until day 84. Flupirtine induced sustained neuroprotection, when delivered up to 9 hours. The latter yielded enhanced neurological recovery that persisted over three months and which was accompanied by enhanced angioneurogenesis. On the molecular level, inhibition of calpain activation was noted, which was associated with increased signal-transducer-and-activator-of-transcription-6 (STAT6) abundance, reduced N-terminal-Jun-kinase and NF-κB activation, as well as reduced proteasomal activity. Consequently, blood-brain-barrier integrity was stabilized, oxidative stress was reduced and brain leukocyte infiltration was diminished. In view of its excellent tolerability, considering its sustained effects on neurological recovery, brain tissue survival and remodeling, flupirtine is an attractive candidate for stroke therapy.

## INTRODUCTION

Following the great advancement of recanalizing therapies in the recent past, which allow to obtain a significant percentage of patients without major deficits by combination of tissue-plasminogen activator-induced thrombolysis and interventional clot removal [1, 2], there remains a major need of establishing restorative therapies for sustained neurological recovery in patients with persistent neurological deficits. To that end, the identification of candidate drugs that are already approved by local authorities is highly attractive because clinical studies may be implemented without time-consuming preclinical safety evaluations.

The triaminopyridine derivative flupirtine is an indirect N-methyl-D-aspartate receptor (NMDAR) antagonist that reduces glutamatergic neurotransmission by activation of inwardly rectifying potassium channels (Kir), thus stabilizing the resting membrane potential and enhancing the Mg^2+^ block [3, 4]. Upon stimulation of neuronal Kv7 potassium channels and subsequent reduction of excessive neuronal action potential generation, flupirtine induces pain relief and muscle relaxation [5-7].

In view of its excellent tolerability, flupirtine has been used for several decades in human patients. During this time, flupirtine gained interest not only as an analgetic but also as a potential neuroprotective compound. Since the 1990-ies, flupirtine has been demonstrated to protect against slowly progressive neuronal degeneration [8-12] and hypoxic-ischemic injury [13-18]. *In vitro* studies suggested that the neuroprotective effects in hypoxia-ischemia are due to prevention of intracellular calcium overload and oxidative stress reduction [14-17].

As a matter of interest, only two studies analyzed flupirtine-induced neuroprotection *in vivo* in models of global or permanent focal cerebral ischemia [13, 16]. In these studies, pre-treatment with flupirtine reduced both neurological impairment and histological brain injury for up to two weeks. In these models, reduction of brain injury was not observed when flupirtine was given after stroke induction. Thus, the therapeutic potential was considered low. Taking into account the excellent tolerability profile of flupirtine, considering the potential impact of NMDAR antagonists in the post-acute ischemic phase, we systematically reevaluated the therapeutic potential of flupirtine after transient focal cerebral ischemia in mice, followed by an analysis of mechanisms involved in both acute neuroprotection and post-acute brain remodeling.

## RESULTS

### Flupirtine induces acute neuroprotection, reduces motor coordination impairment and ameliorates rt-PA-induced toxicity

**Figure 1:**
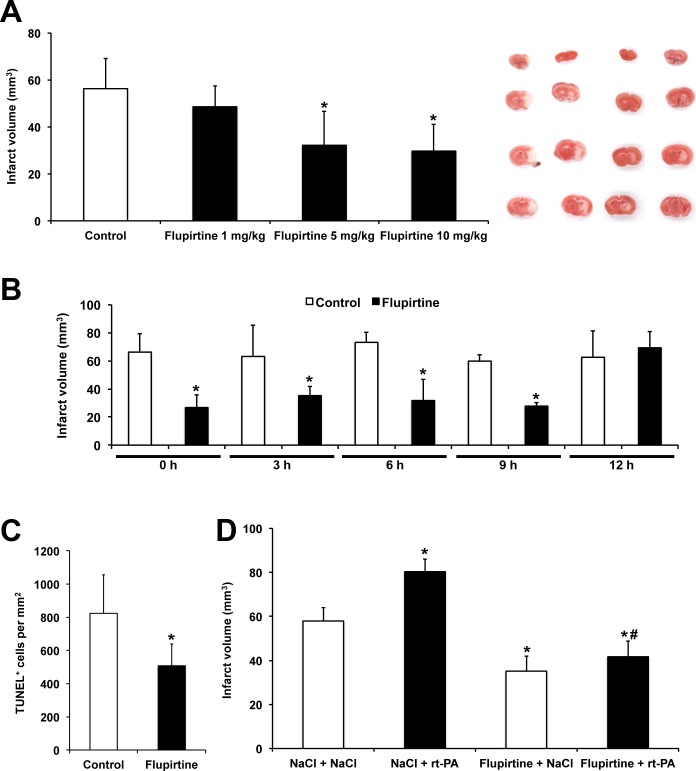
Flupirtine induces acute neuroprotection with a broad therapeutic window **A.** Mice received single intraperitoneal injections of flupirtine at given doses (mg/kg body weight) during post-ischemic reperfusion. Infarct analysis was performed on day 2 using TTC staining. Controls received standard saline only. Representative TTC stainings are shown for each experimental condition in the same order as in the diagram. **B.** In order to assess the potential therapeutic time window, flupirtine (10 mg/kg body weight) was given at the time points given followed by subsequent analysis of infarct volumes on day 2. **C.** Analysis of TUNEL^+^ cells on day 2 after stroke induction. Mice received intraperitoneal treatment with saline (controls) or with flupirtine (10 mg/kg body weight) at 9 h post-stroke. **D.** For analysis of flupirtine-mediated effects on rt-PA-induced brain toxicity, mice intravenously received either rt-PA or NaCl during reperfusion followed by intraperitoneal injections of either flupirtine (10 mg/kg body weight) or NaCl at 9 h post-stroke. Infarct volumes were determined as mentioned afore, i.e. TTC staining was done on day 2. *Significantly different from controls **A.**-**C.** or significantly different from mice that have received intravenous and intraperitoneal injection of NaCl (NaCl/NaCl), *p* < 0.05. #Significantly different from mice that had been treated with rt-PA during reperfusion followed by intraperitoneal treatment with NaCl at 9 h post-stroke, *p* < 0.05.

Before assessing the therapeutic time window dose response experiments were performed, for which flupirtine was injected during reperfusion using a dose between 1-10 mg/kg BW following the protocol from Block et al. who used 5 mg/kg BW [13]. Whereas a dose of both 5 and 10 mg/kg BW reduced infarct volumes on day 2 post-stroke, no effect was observed with 1 mg/kg BW (Figure 1A). Since no signs of toxicity were obvious after treatment with either of the aforementioned doses, future experiments were done using 10 mg/kg BW. Application of the latter proved to be neuroprotective when given no later than 9 h post-stroke (Figure 1B-1C), as shown by infarct volume analysis as well as by assessment of TUNEL^+^ cells on day 2. Interestingly, the same treatment paradigm proved to be successful to reduce rt-PA-mediated acute brain toxicity (Figure 1D), again suggesting possible clinical feasibility of flupirtine.

**Figure 2:**
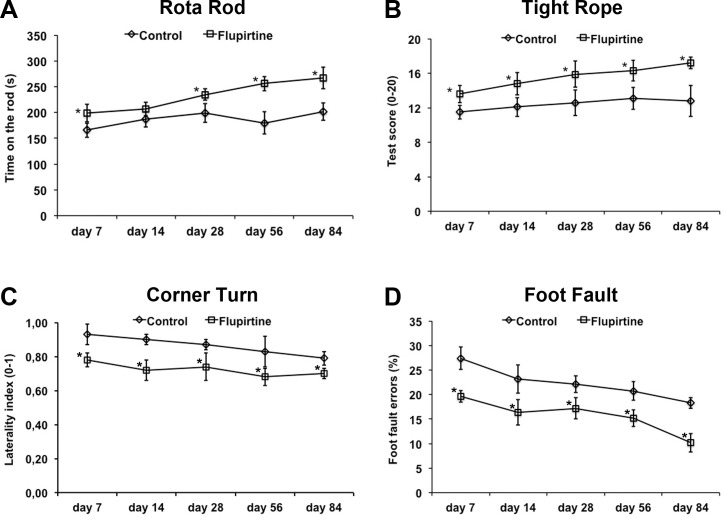
Flupirtine reduces post-ischemic motor coordination impairment Functional recovery upon intraperitoneal treatment with flupirtine (10 mg/kg body weight) 9 h after stroke was assessed using the rota rod **A.,** tight rope **B.,** corner turn **C.,** and foot fault **D.,** test. Control animals received standard saline only. *Significantly different from controls, *p* < 0.05.

Reduction of infarct volumes does not necessarily reflect reduction of motor coordination impairment. Consequently, motor coordination was analyzed for as long as twelve weeks after induction of stroke. Treatment of mice with flupirtine at a dose of 10 mg/kg BW at 9 h post-stroke (which was the experimental paradigm used for the remaining studies) resulted in better test performance in all four behavioral tests when compared to saline controls (Figure 2).

### Flupirtine enhances post-stroke neuroregeneration

**Figure 3:**
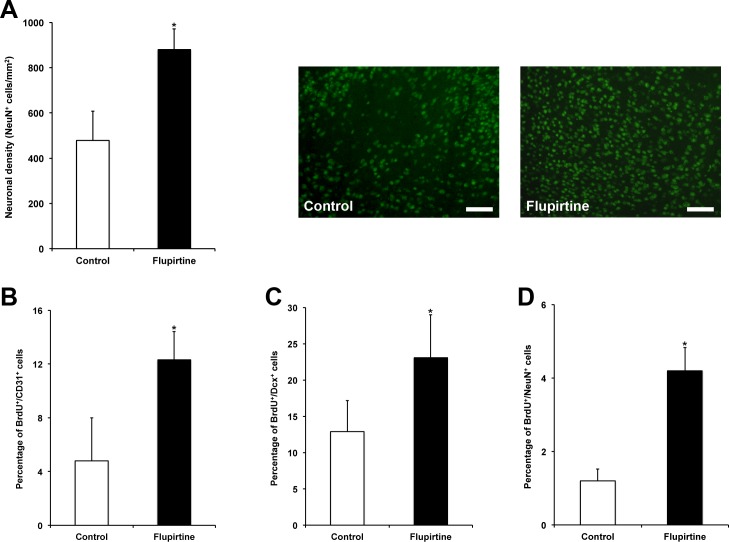
Flupirtine treatment induces sustained neuroprotection and is associated with post-ischemic neuroregeneration **A.** Brain injury was assessed via analysis of neuronal density after NeuN staining on day 84. Representative photos were taken within the lesion site from both control mice (saline only) and flupirtine-treated (10 mg/kg body weight at 9 h post-stroke) mice. Post-ischemic angioneurogenesis was assessed by counterstaining of BrdU^+^ cells with CD31 (**B.** endothelial cell marker), Dcx (**C.** immature neuronal marker) and NeuN (**D.** mature neuronal marker). *Significantly different from controls, *p* < 0.05. Scale bars: 50 μm.

Studying acute neuroprotection is not sufficient in order to assess the therapeutic potential of a drug, which is one reason for negative clinical trials despite positive results in rodent stroke models. As such, brain injury and post-ischemic angioneurogenesis was analyzed three months after focal cerebral ischemia. Brain injury as shown by neuronal density was significantly reduced due to treatment with flupirtine, suggesting that flupirtine when given no later than 9 h induces stable and sustained neuroprotection in our model (Figure 3A). In this context, analysis of post-ischemic angioneurogenesis revealed significantly increased numbers of new-born CD31^+^ cells (Figure 3B; endothelial cell marker), Dcx^+^ cells (Figure 3C; immature neuronal marker) and NeuN^+^ cells (Figure 3D; mature neuronal marker), albeit the latter was low in number. Consequently, flupirtine treatment is not only associated with neuroprotection but also with increased post-ischemic angioneurogenesis, which might affect each other mutually.

### Flupirtine induces stabilization of the blood-brain-barrier and reduction of oxidative stress and intracerebral inflammation

**Figure 4:**
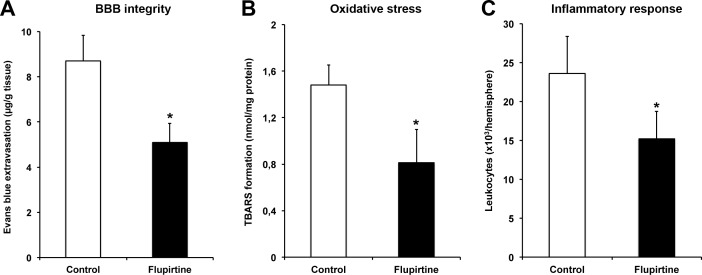
Flupirtine induces stabilization of the blood-brain-barrier and reduction of oxidative stress and intracerebral inflammation Mice were treated with either flupirtine (10 mg/kg body weight) or standard saline (control) at 9 h post-stroke followed by sacrifice of animals 48 h after stroke induction. Assessment of blood-brain-barrier integrity was performed by measurement of Evans blue extravasation, which was intravenously applied 2 h before sacrifice of animals **A.** Oxidative stress was indirectly analyzed in ischemic hemispheres by determination of thiobarbituric acid reactive substance (TBARS) formation at 48 h post-ischemia **B.** Inflammatory responses within the ischemic lesion site were assessed using quantitative analyses of absolute amounts of CD45^+^ leukocytes 48 h after stroke **C.**. *Significantly different from controls, *p* < 0.05.

Although flupirtine is known to act via indirect antagonism of NMDAR, effects on blood-brain-barrier (BBB) integrity and reduction of post-ischemic oxidative stress as well as inflammation within the ischemic lesion site have not been studied, yet. Consequently, we assessed BBB integrity as well as oxidative and inflammatory stress at 48 h after stroke induction in brain lysates. Acute treatment with flupirtine 9 h post-stroke significantly reduced extravasation of Evans blue when compared to controls, suggesting that flupirtine enhances BBB integrity (Figure 4A). Likewise, treatment with flupirtine reduced oxidative stress within the ischemic lesion site (Figure 4B). Intracerebral inflammatory response as analyzed by flow cytometry was significantly reduced after treatment with flupirtine when compared to controls (Figure 4C), suggesting that flupirtine reduces both post-ischemic oxidative stress and inflammation.

### Flupirtine inhibits calpain-dependent degradation of STAT6 resulting in reduced proteasomal activity

Flupirtine reduces stroke-induced calcium increase [16]. The latter is critically involved in pro-injurious signaling pathways involving calpains as well as c-Jun-N-terminal kinases (JNK) and nuclear factor-κB (NF-κB) [19-21]. Cerebral ischemia has been shown to reduce levels of the transcription factor signal-transducer-and-activator-of-transcription-6 (STAT6) [22], which is also degraded by calpain [23]. Recently, STAT6 has been demonstrated to inhibit JNK and NF-κB signaling pathways under inflammatory conditions outside the CNS [24]. We therefore analyzed whether or not flupirtine affects these cell signaling pathways after induction of cerebral ischemia.

**Figure 5:**
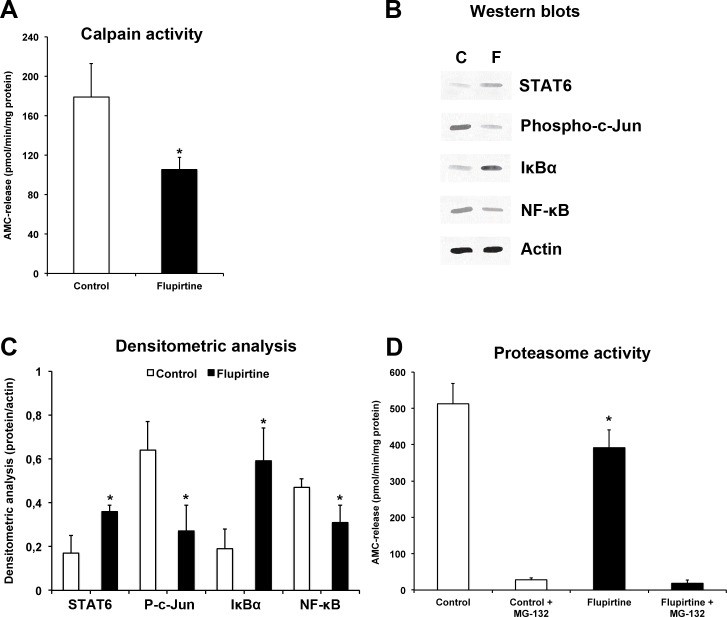
Flupirtine exerts acute neuroprotection via modulation of JNK and NF-κB signaling pathways resulting in reduced proteasomal activity Mice were treated with either flupirtine (10 mg/kg body weight) or NaCl (control) 9 h after stroke induction. Mice were sacrificed 48 h post-stroke. **A.** Calpain activity was measured in ischemic hemispheres using Suc-Leu-Leu-Val-Tyr-AMC as protease substrate in the presence of calcium. **B.** Western blotting using ischemic hemispheres for detection of STAT6, NF-κB, IκBα and phosphorylated c-Jun (“Phospho-c-Jun”) at 48 h post-stroke in controls (“C”) and flupirtine-treated mice (“F”). **C.** Densitometric analysis of (B). **D.** Determination of the chymotrypsin-like activity of the proteasome using Su-Leu-Leu-Val-Tyr-AMC. Since the latter is not specific for detection of proteasomal activity, some samples were incubated with the proteasome inhibitor MG-132 (1 μM). *Significantly different from controls, *p* < 0.05.

Analysis of calpain activity 48 h post-stroke revealed significantly increased protease activity in control animals, whereas treatment with flupirtine reduced calpain activity (Figure 5A). In this context, induction of cerebral ischemia resulted in activation of the JNK (expressed as abundance of phosphorylated c-Jun) and NF-κB pathway, which is controlled by its physiological inhibitor IκB-α (Figure 5B-5C). Expression of the latter was reduced in controls (Figure 5B-5C). Treatment with flupirtine, however, reversed the aforementioned effects, i.e. both JNK and NF-κB signaling pathways were inhibited by flupirtine. Interestingly, stroke-induced reduction of STAT6 was significantly reversed by flupirtine as well (Figure 5B-5C). Since NF-κB signaling is associated with activation of the proteasome, which is one key component in developing ischemic brain injury [25], chymotrypsin-like activity was measured in brain lysates. Two days after stroke, proteasome activity was significantly reduced when compared to controls (Figure 5D). These data suggest that flupirtine indirectly exerts post-ischemic neuroprotection via reduction of calcium-induced activation of JNK and NF-κB signaling pathways associated with reduced proteasomal activity.

## DISCUSSION

The present study analyzed the therapeutic potential of flupirtine and its underlying mechanisms during an observation period of three months after induction of stroke. We show that flupirtine induces sustained neuroprotection and neuroregeneration in a dose-dependent manner when given no later than nine hours post-stroke. In this context, flupirtine reduces calcium dependent calpain activation followed by restoration of STAT6 and subsequent inhibition of both JNK and NF-κB pathways. Inhibition of the latter is associated with post-stroke activation of the proteasome and thus helps reduce post-ischemic brain injury.

Excitotoxicity after cerebral ischemia has long been in focus of experimental stroke research. Yet, the majority of neuroprotective trials have failed in clinical settings due to narrow time window, side effects and low number of patients as well as inappropriate quality standards [26]. Despite recent reports on liver toxicity of flupirtine [27], the therapeutic potential of flupirtine is intriguing taken into account the overall low frequency of side effects with no clinically relevant drug interactions [7] and both high bioavailability after oral/rectal administration as well as a high distribution volume into virtually all extravascular and intravascular compartments [6]. Indeed, derivatives of flupirtine such as retigabine have been approved for treatment of CNS disorders such as epilepsy [28], again underlying the therapeutic potential of flupirtine and derivatives for treatment of ischemic stroke.

Stimulation or inhibition of NMDAR remains a double-edged sword. As such, stimulation of NMDAR can induce both cell survival or cell death depending on NMDA concentrations. Previous *in vitro* data suggest that modest stimulation or inhibition of NMDAR promote neuronal survival, whereas high concentrations of NMDA or appropriate inhibitors induce cell death [29, 30]. Likewise, complete inhibition or knockout of the NMDAR induces cell death during ontogenesis of the brain [31, 32]. Hence, the hypothesis on extrasynaptic versus synaptic stimulation of NMDAR has been suggested with stimulation of synaptic NMDAR to induce pro-survival cascades such as ERK and stimulation of extrasynaptic receptors to induce death signal cascades [21, 26]. Neuroprotection due to flupirtine treatment might thus indirectly inhibit extrasynaptic NMDAR, contributing to neuronal survival. The latter in turn is a consequence of decreased calcium concentrations [16].

Calcium is one key factor in excitotoxic ischemic brain injury, among which the activation of calpains is crucial. As such, an NMDA-induced increase in intracellular calcium with subsequent activation of calpain is the most important signaling pathway contributing to post-ischemic proteolysis and ultimately cell death [33, 34]. Consequently, calpain activity is significantly induced by treatment with flupirtine in our study. Among a great deal of substrates to calpain degradation, STAT proteins such as neuroprotective STAT6 are critically involved [23]. STAT6 expression has recently been shown to be reduced within the ischemic rodent brain after induction of stroke [22], and treatment with flupirtine reverses this effect. Moreover, STAT6 has been described to inhibit JNK and NF-κB signaling pathways [24], which are critically involved in progression of ischemic brain injury [20, 35-37]. Treatment with flupirtine inhibits these pathways and reduces proteasomal activity, which in turn regulates NF-κB signaling via degradation of IκB-α [38]. Reduced proteasomal activity after treatment with flupirtine is thus one critical component contributing to tissue injury during cerebral ischemia.

Although the aforementioned mechanisms underlying flupirtine-induced neuroprotection are most likely not exclusive by nature of indirect NMDAR antagonism, they seem to significantly have an impact on readout parameters of assessing post-ischemic brain injury. In this context, subacute treatment with flupirtine did not only reduce infarct volumes and stabilized BBB integrity but also reduced the inflammatory response and oxidative stress within the ischemic lesion site, most likely due to reduced proteasome-NF-κB and JNK signaling. Of note, the dosage used for the present study was of clinical relevance where dosages of some hundred milligrams are in order to achieve pain reduction [7], albeit the present work did not study effects of retard pills of flupirtine. More importantly, these acute and profound effects were associated with sustained and stable neurological recovery for at least three months involving enhanced post-ischemic angioneurogenesis. The herein described and sustained neuroprotection is, however, in contrast to previous reports on flupirtine-induced neuroprotection after experimental stroke [13, 16]. These studies demonstrated reduced post-ischemic brain injury for up to two weeks when flupirtine was applied before induction of stroke, but failed to do so when flupirtine was injected after stroke induction. On the contrary, the present study successfully demonstrates flupirtine-induced neuroprotection after treatment no later than nine hours post-stroke. The reason for this discrepancy might be due to different animal strains (Wistar rats and NMRI mice) and stroke models (transient global cerebral ischemia and permanent focal cerebral ischemia) used. Consequently, further pre-clinical studies are in order to further validate the therapeutic potential of flupirtine.

In conclusion, subacute treatment with flupirtine is an intriguing adjuvant tool next to thrombolysis inducing long-term neuroprotection and enhanced neurological recovery after transient focal cerebral ischemia. In light of already been approved by local authorities in Europe as an analgesic, adjuvant treatment of stroke with flupirtine next to thrombolysis appears to be a feasible tool in stroke treatment. However, further pre-clinical studies are in order to further evaluate the therapeutic potential of flupirtine before the path can be set for clinical trials.

## MATERIALS AND METHODS

### Experimental paradigm

All experiments were performed according to local authorities. Male C57BL6 mice (Harlan, Germany; 22-25 g each) were kept under circadian rhythm and had free access to food and water. Animals were strictly randomized and studies were blinded to both experimenters and analysts. Observation period was up to 84 days after induction of cerebral ischemia. All mice received peri-procedural analgesic therapy applying single subcutaneous injection of carprofen (4 mg/kg body weight (BW)) that was continued on the three subsequent days or end of experiment, respectively. The number of animals used for statistical analysis including survival rates is given in Table 1.

**Table 1:** Experimental animals used for statistical analysis

	Day 2	Day 84
**TTC analysis (Reperfusion treatment)**		
Control	*n =* 8 (100.0%)	
Flupirtine 1 mg/kg	*n =* 7 (77.8%)	
Flupirtine 5 mg/kg	*n =* 7 (87.5%)	
Flupirtine 10 mg/kg	*n =* 8 (100.0%)	
**TTC analysis (Time window)**		
Control 0 h	*n =* 7 (100.0%)	
Flupirtine 0 h	*n =* 8 (88.9%)	
Control 3 h	*n =* 8 (100.0%)	
Flupirtine 3 h	*n =* 7 (100.0%)	
Control 6 h	*n =* 8 (80.0%)	
Flupirtine 6 h	*n =* 7 (100.0%)	
Control 9 h	*n =* 7 (100.0%)	
Flupirtine 9 h	*n =* 7 (100.0%)	
Control 12 h	*n =* 8 (88.9%)	
Flupirtine 12 h	*n =* 8 (100.0%)	
**TTC analysis (rt-PA approach)**		
NaCl + *N* aCl	*n =* 7 (100.0%)	
NaCl + rt-PA	*n =* 7 (100.0%)	
Flupirtine + NaCl	*n =* 7 (100.0%)	
Flupirtine + rt-PA	*n =* 8 (88.9%)	
**Protease activities**		
Control	*n =* 5 (100.0%)	
Flupirtine	*n =* 5 (100.0%)	
**Evans blue measurement**		
Control	*n =* 4 (100.0%)	
Flupirtine	*n =* 4 (100.0%)	
**TBARS formation**		
Control	*n =* 4 (100.0%)	
Flupirtine	*n =* 4 (100.0%)	
**Western blotting**		
Control	*n =* 5 (83.3%)	
Flupirtine	*n =* 5 (100.0%)	
**Flow cytometry**		
Control	*n =* 7 (100.0%)	
Flupirtine	*n =* 7 (100.0%)	
**IHC / Behavioral tests**		
Control	*n =* 5 (100.0%)	*n* =15 (93.8%)
Flupirtine	*n =* 5 (100.0%)	*n* =13 (81.3%)

All animals had free access to food and water and were kept under circadian rhythm. Experimental procedures were blinded to experimenters and analysts. Male C57BL6 mice were used for all experiments. Mice were treated intraperitoneally with either flupirtine or NaCl (controls) with some of which receiving additional intravenous treatment with rt-PA or NaCl during reperfusion. Mice were sacrificed on days 2 or 84 after induction of stroke. Values given represent final number of animals that were used for statistical analysis of each experimental group with survival rates indicated in brackets. Abbreviations: IHC: immunohistochemistry, TBARS: thiobarbituric acid reactive substances, TTC: 2,3,5-triphenyltetrazolium chloride.

Experimental mice received intraperitoneal (i.p.) treatment with either flupirtine or normal saline. Treatment was performed during reperfusion, 3 h, 6 h, 9 h and 12 h post-stroke. In dosage finding experiments, flupirtine dosages ranged between 1 mg and 10 mg per kg BW. The majority of experiments were performed using a flupirtine dose of 10 mg per kg BW at 9 h after stroke induction. For some experiments, intravenous injection of rt-PA (10 mg/kg BW) or saline during reperfusion via cannulation of the femoral vein was performed.

### Induction of transient focal cerebral ischemia

Cerebral ischemia was induced as previously described [39]. Briefly, mice were set under anesthesia using a volatile mixture of isoflurane (1.5%), oxygen (30%) and nitrogen (remainder). Using the thread occlusion model, a silicon-coated nylon filament (Doccol, USA) was inserted into the left common carotid artery and gently pushed upwards towards the left middle cerebral artery (MCA). The thread was kept *in situ* for 30 min under constant Laser Doppler flow control using a probe (Perimed, Sweden) that was placed on the intact skull covering the left MCA territory. A Laser Doppler flow drop towards 30% of its initial arbitrary value was regarded as successful induction of stroke. After thread removal, blood flow was observed for an additional 15-min period in order to guarantee reperfusion.

### Assessment of brain injury and post-ischemic angioneurogenesis

Acute brain injury was assessed on day 2 post-stroke via determination of infarct volumes. Brains were removed and cut into slices of 2 mm each. Thereafter, slices were stained with 2,3,5-triphenyltetrazolium chloride (TTC, 2%) followed by a computer-based analysis of ischemic lesion volumes using ImageJ software. For immunohistochemical analyses, mice were sacrificed and transcardially perfused with saline and 4% paraformaldehyde [40]. Brains were removed and cryostat sections of 20 μm each were generated. Three sections per animal were used for tissue processing for each staining. Quantitative analyses were performed using well-defined regions of interests (ROIs) at AP + 0.14 mm, ML ± 1.5-2.25 mm and DV - 2.5-3.25 mm. Four ROIs per section were used for statistical analysis.

In order to further assess acute brain injury next infarct volume analysis, TUNEL staining on day 2 post-stroke was performed. Sections were incubated with proteinase K (7 min, 37°C), followed by exposure to TdT enzyme reaction according to the manufacturer's manual (Roche, Germany). Thereafter, sections were repeatedly washed followed by staining with a streptavidin-Alexa488-conjugated secondary antibody (2 h at room temperature (RT); Abcam, Germany).

Further immunohistochemical stainings were done with incubation periods of 18 h (4°C) for primary antibodies followed by repeated washing steps and an additional incubation with secondary antibodies for 1 h at RT. Sustained neuroprotection was assessed via analysis of neuronal density using NeuN staining on day 84 with a mouse monoclonal anti-NeuN primary antibody (1:1,000; Millipore, UK) and a goat anti-mouse Alexa 488 (1:100; Jackson ImmunoResearch, UK) secondary antibody. For assessment of post-ischemic angioneurogenesis, mice received daily single injections of bromodeoxyuridine (BrdU; 50 mg/kg BW) from days 8 to 40. The primary antibodies were as follows: monoclonal mouse anti-BrdU antibody (1:400; Roche, Switzerland), monoclonal rat anti-BrdU antibody (1:400; Abcam, UK), goat polyclonal anti-doublecortin antibody (1:50; Santa Cruz Biotechnology, Germany), monoclonal rat CD31 (1:200, BD Biosciences, Germany) and mouse monoclonal anti-NeuN antibody (1:1,000; Millipore). The following secondary antibodies were used: goat anti-mouse Cy-3 (1:400; Dianova, Germany), goat anti-rat Alexa 594 (1:400; Dianova), donkey anti-goat Alexa 488 (1:250; Invitrogen, Germany), goat anti-mouse Alexa 488 (1:100; Jackson ImmunoResearch), and goat anti-rat Alexa 488 (1:250; Invitrogen).

### Behavioral tests

Assessment of post-ischemic neurological impairment was done using well-defined behavioral tests such as the rota rod, tight rope, corner turn and foot fault test [39]. Animals were trained 1-2 days before induction of stroke in order to ensure proper test performance. The tests were done on days 7, 14, 28, 56 and 84 after stroke induction. As for the corner turn test, the mouse was placed into an apparatus consisting of two vertical boards forming an angle of 30°. Test parameter was the mouse's side chosen to leave the corner once it made contact to the boards with its whiskers. Ten trials were performed on each day. The laterality index was calculated as follows: (number of left turns – number of right turns)/10 with indices approximating 1.0 demonstrating severe motor coordination deficits. Rota rod test performance was analyzed using a rota rod with accelerating velocity (4-40 rpm). Maximal velocity was achieved after 260 s and maximal testing time was 300 s. The time until the animal dropped was recorded and used for statistical analysis. Mice were tested twice per time point and means were calculated. Likewise, the tight rope test was performed twice per time point for which the animal was placed in the middle of a 600 mm long rope that was attached to a platform on either side. Two parameters were assessed, i.e. whether or not the animal reached the platform and the time spent on the rope. Maximal testing time was 60 s. Test results are given according to a validated score from 0 (min) to 20 (max). The foot fault test was performed using an elevated steel grid on which the mouse was placed. Test parameters include the total number of steps for each forelimb during the process of moving forward. From the total amount of right forelimb steps, the relative percentage of foot fault errors (i.e. when animals misplaced their forelimbs) for the right forelimb was calculated. Data is given as percentage of foot fault errors for the right impaired forelimb referring to the total amount of right forelimb steps.

### Analysis of blood-brain-barrier integrity

Integrity of blood-brain-barrier (BBB) was analyzed 48 h after induction of stroke as previously described [41]. Briefly, Evans blue (2%; 2 mL/kg BW) was intravenously injected 2 h before sacrifice of animals. Left hemispheres were weighed, homogenized in 2 mL of 50% trichloroacetic acid and used for photometric analysis of Evans blue extravasation. A luminescence spectrophotometer (excitation 620 nm and emission 680 nm) was used for measurements. Values are given as (μg) Evans blue dye per (g) tissue.

### Analysis of TBARS formation

The extent of post-ischemic burst within the ischemic hemisphere was indirectly assessed at 48 h post-stroke using measurement of peroxidized fatty acids of phospholipids. During this process, thiobarbituric acid reactive substances (TBARS) such as malondialdehyde (MDA) are formed. The latter reacts with thiobarbituric acid giving rise to a chromogenic compound, which can be photometrically measured at 532 nm [42, 43]. Data are given as MDA equivalents using 1,1,3,3-tetramethoxypropane as standard.

### Flow cytometry analysis

Flow cytometry analysis for determination of absolute amounts of leukocytes (CD45^+high^) within the ischemic hemisphere was previously described [40, 44]. Briefly, mice were sacrificed 48 h after stroke induction followed by perfusion with PBS and preparation of left ischemic hemispheres that were then homogenized, centrifuged and resuspended in 30% percoll (GE Healthcare, Sweden) that included 70% percoll below and centrifugation of samples for 20 min at 2,400 rpm at RT. After washing, cells were incubated with a rat anti-CD45 (BioLegend, Germany) antibody for 30 min (4°C). Countbright counting beads (Invitrogen, Carlsbad, CA, USA) were included for quantitative analysis.

### Determination of protease activities

Measurement of both calpain and proteasome activities were done at 48 h post-stroke as previously described [43, 45]. Briefly, protease activities were fluorimetrically determined in brain homogenates (100 mmol/L Tris-HCl, 145 mmol/L NaCl, 10 mmol/L EDTA and 0.5% Triton X-100 at pH 7.4) using a fluorescence microtiter plate reader at 37°C with λexc.=355 nm and λem.=460 nm. Thereafter, lysates were used for either determination of calpain or proteasome activity. For detection of calpain activity, samples were incubated with a buffer of 100 mM Tris-HCl, 145 mM NaCl and 10 mM EDTA at pH 7.4 that was supplemented with 50 μM of Suc-Leu-Leu-Val-Tyr-AMC (Bachem, Switzerland) as reaction substrate (with or without addition of 10 mM Ca^2+^). Proteasomal activity (i.e., chymotrypsin-like activity) was performed using a measurement buffer consisting of 50 mmol/L Tris, 20 mmol/L KCl, 1 mmol/L magnesium acetate, 2 mmol/L dithiothreitol, 1 mmol/L leupeptin, 1 mg/mL aprotinin (Sigma-Aldrich, Taufkirchen, Germany) and 1 mmol/L PMSF (Merck, Darmstadt, Germany). Suc-Leu-Leu-Val-Tyr-AMC served again as substrate. As Suc-Leu-Leu-Val-Tyr-AMC is not specific for proteasome activity, the proteasome inhibitor MG-132 (1 mmol/L; Sigma-Aldrich) was added to some samples. Protease activities are given in picomol per min per milligram protein.

### Western blotting

Blotting was performed 48 h after stroke induction using ischemic left hemispheres. The latter were homogenized in lysis buffer (50 mmol/L Tris, pH 8.0, 150 mmol/L NaCl, 1% Triton X-100, protease inhibitors) followed by centrifugation steps. The supernatants were used for SDS-PAGE electrophoresis where equal amounts of protein (40 μg) were loaded onto 12% polyacrylamide gels. Proteins were transferred onto membranes, which were incubated overnight (4°C) with the following primary antibodies: rabbit polyclonal anti-pSTAT6 (Tyr641; Abcam, UK), mouse monoclonal anti-NF-κB p65 (Santa Cruz Biotechnology, Germany), rabbit polyclonal anti-phospho-c-Jun (Santa Cruz Biotechnology), mouse monoclonal anti-IκB-α (Santa Cruz Biotechnology). Membranes were then incubated with a peroxidase-coupled goat anti-rabbit or goat anti-mouse secondary antibody (Santa Cruz Biotechnology), washed several times, immersed in ECL solution and exposed to ECL-Hyperfilm. Thereafter, membranes were scanned and used for densitometric quantitative analysis of protein abundance.

### Statistical analysis

Data presented in this work are given as means ± standard deviation (SD). The Student's t test was used for comparison between two groups. For comparison between more groups a one-way analysis of variance (ANOVA) followed by Tukey's post hoc tests was done. A *p*-value of < 0.05 was regarded statistically significant.
